# Characterization of permanent deformation properties of densely-compacted unbound granular materials subjected to cyclic loading

**DOI:** 10.1038/s41598-023-30635-7

**Published:** 2023-03-06

**Authors:** Gang Liu, Mingzhi Zhao, Qiang Luo, Rui Lu

**Affiliations:** 1 School of Architecture and Civil Engineering, Xihua University, Chengdu, 610039 Sichuan China; 2 Institute of Geotechnical Engineering, Xihua University, Chengdu, 610039 Sichuan China; 3 MOE Key Laboratory of High-Speed Railway Engineering, Southwest Jiaotong University, Chengdu, 610031 Sichuan China

**Keywords:** Civil engineering, Mechanical engineering

## Abstract

To investigate the long-term deformation properties of unbound granular materials (UGM) that are ordinarily adopted to construct subgrade for high-speed railway, a series of medium-sized cyclic triaxial tests were performed to obtain the relationship between permanent strain and loading cycles under different cyclic stress levels. Moreover, DEM analysis was conducted for the samples to reveal the deformation mechanism and verify the strain developing tendency. It is found that the UGM samples present different long-term deformation properties under different cyclic stress levels. As cyclic stress increases, the permanent strain of UGM sample transfers from rapid stabilization to tardy stabilization, then to tardy failure and finally to rapid failure. Furthermore, the exponent in a power law function was selected as the critical indicator of deformation developing tendency. With the exponent obtained precisely in accordance with the strain rate, the deformation tendency can be analyzed quantitatively. Finally, the characteristics of interparticle force chains induced by different cyclic stress levels were obtained by DEM analysis, which provided evidences for the classification of long-term deformation properties of UGM samples. The achievements have guiding significance for the design of subgrade of both ballasted and unballasted high-speed railway.

## Introduction

Subgrade is a kind of soil structure that is typically filled with native materials and compacted to a targeted density to provide foundational support for the railway tracks. Since subgrade directly suffers the dynamic load transferred from the track structure, it is usually constructed by unbound granular materials (UGMs), in whole or in part, to disperse the dynamic load and transfer downward^1^. With the rise of traffic volume and improvement of operation speed in railway engineering, UGMs are increasingly adopted to construct subgrade in modern railway construction due to their excellent engineering properties. As the uppermost substructure, the UGM functional layer should have prominent resistance to deformation under dynamic load. Especially, the accumulated permanent deformation of subgrade, which directly deteriorates the smoothness and stability of rail track, should be controlled strictly^2,3^. However, since the tolerance of post-construction deformation of high-speed railway is required and specified at millimeter-scale, to evaluate the exact deformation value for the soil structure precisely is very difficult^4,5^. Therefore, it is of great significance to investigate the long-term deformation properties of UGM samples to gain the understanding of service performance for subgrade.

Currently, the long-term deformation properties of UGM fillings in subgrade can be greatly improved by optimizing the quality of fillings and enhancing the degree of compaction. It is specified in Japan that the surface layer of subgrade should be constructed layer by layer with graded gravel and asphalt concrete to improve the service performance^1^. While in Germany, it is required that the fillings in subgrade should be primarily coarse-grained soils with degree of compaction larger than 97%^6^. Furthermore, well-graded angular gravel is required to construct the surface layer of subgrade in China^1^. Even though the design criteria of subgrade for high-speed railway have already been put forward in some countries, permanent deformation properties of the UGM fillings are not sufficiently taken into consideration in these codes. This may result in conservative engineering measures in railway construction. For example, since ballasted track could be raised to the original location by replenishing new ballast once post-construction deformation occurs, it should have requirements of UGM fillings that are different from the unballasted one from the view of permanent deformation. Thus, the long-term deformation properties of UGM fillings are necessary to be evaluated quantitatively to provide guidance to the design criteria of subgrade.

To evaluate the permanent deformation of UGM quantitatively, two kinds of technical approaches have been put forward in the previous literatures. One is to develop analysis model to predict the relationship between permanent deformation and loading cycles^7–13^. This approach has a purpose of calculating the exact deformation value of the UGM fillings. The other emphasizes to evaluate the developing tendency of permanent deformation quantitatively^4,5,14,15^. In this framework, the deformation rate is plotted against loading cycles to demonstrate the developing tendency so that different deformation stages can be categorized. Since the definite value of millimeter-scale deformation is difficult to predict accurately, the latter is more popular and recognized.

Several classification methods for the developing properties of permanent deformation have been recommended previously. The permanent deformation of London clay and Chengdu clay were plotted against loading cycles respectively under different cyclic amplitudes by performing triaxial test^16,17^. The deformation curves were categorized into the generative pattern and degenerative one in terms of loading amplitude, which corresponds to ever-increasing permanent deformation and converging one, respectively. It is remarkable that two kinds of developing tendency states were identified to depict the developing properties of permanent deformation. Moreover, 100 groups of triaxial tests were performed on graded gravel and sandy gravel at different cyclic amplitudes by Werkmeister^5^. The developing pattern of permanent strain has been divided into three categories in accordance with the relationship between strain rate and loading cycles, i.e., plastic shakedown, plastic creep and incremental collapse. Plastic shakedown represents that strain rate converges to zero in just a few loading cycles, and permanent strain levels off rapidly in the initial loading process. Reversely, incremental collapse implies that strain rate increases with loading cycles, and the sample collapses momentarily due to the ever-increasing permanent strain. Plastic creep is a transitional status, in which the strain rate of the gravel sample decreases initially with loading cycles, and finally levels off. However, the permanent strain increases continuously with loading cycles and finally leads to breaking down of the sample. The difference between the accumulated permanent strains at the 3000th and 5000th loading cycles, i.e. Δ*ε*_*p*,5000_ − Δ*ε*_*p*,3000_, were proposed by Werkmeister as the criterion to classify plastic shakedown, plastic creep and incremental collapse ranges, as presented in Table 1.

**Table 1:** The shakedown and creep limits determined by Δ*ε*_*p*,5000_ − Δ*ε*_*p*,3000_ from different studies.

No	Plastic shakedown limit	Plastic creep limit	Filling position	Literature
1	4.5 × 10^–5^	4.0 × 10^–4^	Pavement	Werkmeister^5,15^
2	6.0 × 10^–5^	6.0 × 10^–4^	Pavement	Gu et al.^22^
3	1.0 × 10^–5^	8.0 × 10^–5^	Subgrade	Wang and Zhuang^21^

Similarly, Minassian also classified the developing properties of permanent deformation of UGMs into three states, i.e., stable, critical and unstable states^18^. Nevertheless, the definition of critical state is ambiguous and deserves further discussion. Besides, Hoff et al. believed that the deformation tendency of UGM samples evolves in three stages in terms of loading amplitude^19^. The first stage is that only elastic strain develops on the condition of small cyclic loading amplitude. As loading amplitude increases, permanent strain emerges but finally levels off to a steady state. When loading amplitude rises to a relatively high level, permanent strain is accumulated and finally results in sample collapse. In addition, by conducting a series of model tests, Liu et al. claimed that the developing tendency of permanent deformation for UGM samples can be grouped into rapid stabilization, tardy stabilization, tardy failure and rapid failure^20^. Wang and Zhuang performed a series of dynamic triaxial tests on subgrade materials under different loading frequencies, confining pressures and cyclic stress ratios^21^. They pointed out that the Werkmeister’s boundaries are also not suitable for the tested crushed limestone aggregate. With this idea in mind, new boundaries were proposed for the subgrade limestone material, as shown in Table 1. Besides, Gu et al. also proposed different shakedown boundaries for Texas pavement materials, which are also listed in Table 1^22^.

As mentioned above, the two ultimate permanent strain developing states, the stable one corresponding to low cyclic amplitudes and the unstable one corresponding to high amplitudes, exactly exist. However, when the loading amplitude stays moderate, whether the intermediate state will lead the sample towards steady or destructive still deserves disputable. Besides, the shakedown and creep limits determined by Δ*ε*_*p*,5000_–Δ*ε*_*p*,3000_ vary significantly with material type, size gradation and test conditions. It is quite difficult to classify the long-term deformation properties of granular materials under different cyclic stress levels by only calculating Δ*ε*_*p*,5000_ − Δ*ε*_*p*,3000_, since the choices of these two relevant strains (strains at the 3000th and 5000th loading cycles) are arbitrary to some extent. In this study, medium-sized triaxial tests were performed on UGM samples with different degrees of compaction under different confining pressures. A power law function with a negative exponent was introduced to depict the relationship between the permanent strain rate and loading cycles, and the power exponent was verified to be able to categorize the developing tendency of permanent strain precisely. With the power exponent as the criterion, the transitional state can be further divided into tardy stabilization status and tardy failure status. Finally, the long-term deformation mechanisms of UGM samples were revealed by discrete element analysis, which further verify the reasonability of the grouping method for deformation properties of UGMs put forward in this study. This classification conception is of great significance for the design of subgrade for ballasted and unballasted rail track in terms of permanent deformation.

## Materials and methods

### Test materials

**Figure 1:**
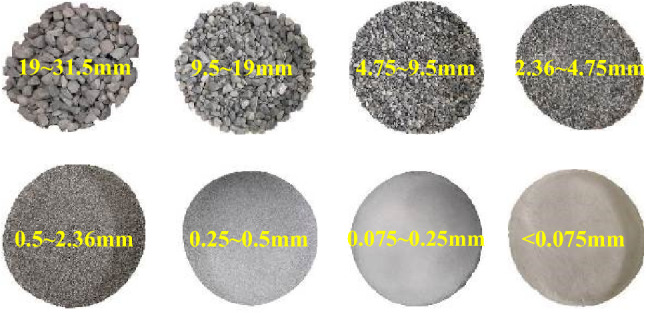
Eight groups of soil particles with different grain sizes.

**Figure 2:**
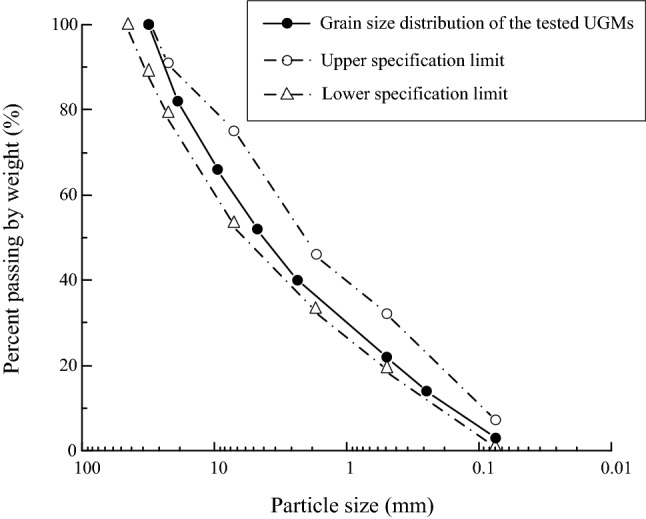
Grain size distribution of the UGM sample in this study.

Unbound granular materials (UGMs) adopted in this study is a kind of graded gravel that originate from quartz stone and sandstone. The UGMs were hand-washed by tap water to remove particles finer than 0.075 mm. After washing, the granular particles were taken to the oven and dried for 4 h at 105 °C, obtaining the pure graded gravel particles. Then, the particles were classified into 8 groups in accordance with particle size, as shown in Fig. 1. The particles with different sizes were mixed in appropriate proportion to obtain the tested UGMs that fulfill the requirements of subgrade fillings specified in *Code for Design of High Speed Railway* (TB 10621-2014)^1^. The grain size distribution curve of the prepared UGM is demonstrated in Fig. 2. As can be seen, the UGM in this study are suitable to be adopted as the fillings of the surface layer of subgrade.

### Sample preparation

Before sample preparation, modified Proctor compaction test has been performed on the UGM to investigate its compaction properties. The Proctor cylinder has a volume of 2103.9 cm^3^, with 152 mm in diameter and 116 mm in length. A drop hammer that weighs 4.5 kg is selected to perform the modified Proctor compaction test. During compaction, the hammer is dropped from a height of 457 mm every time to compact the graded gravels in the Proctor cylinder. By performing compaction test, the maximum dry density of the UGM was measured to be 2.43 g/cm^3^, and the optimum water content was 4.08%. Since the triaxial samples with degrees of compaction (DoC) of 0.95 and 1.00 were intended to be prepared, the UGM were oven-dried for 24 h and weighed to prepare triaxial samples with the targeted DoC. After that, the UGM was mixed with suitable amount of de-aired water to reach the optimum water content (OWC) state and stored in the humidor for 24 h to homogenize the moisture.

According to Code for Soil Tests of Railway Engineering (TB10102-2010)^23^, the method of triaxial samples compacted at different layers was used in this study. The triaxial samples were intended to be 150 mm in diameter and 300 mm in height. The UGM at OWC state were then divided into five equal proportions to prepare the sample in five layers. Each layer remains the same in both height and weight. The triaxial UGM samples were compacted in a compaction container that is composed of three-way split former. During compaction, each equally divided part of UGM was placed into the container and compacted by a Proctor hammer. For each tamping cycle, the hammer was set at the same initial height to ensure the same compaction energy each time. After tamped to the desired height, the surface of the layer was scraped to a depth of 2 mm to ensure good interlocking with the adjacent layers^2,24^. Since the lower layers received more compaction energy compared with the upper layers, this sample preparation method may inherently cause nonuniformity along depth. However, this error can be neglected due to the same effect for each sample. After compaction, a rubber membrane was spread to enclose the sample, and silicone grease was smeared across the top and bottom of the specimen to minimize the friction with the top cap and bottom pedestal.

### Test program

**Figure 3:**
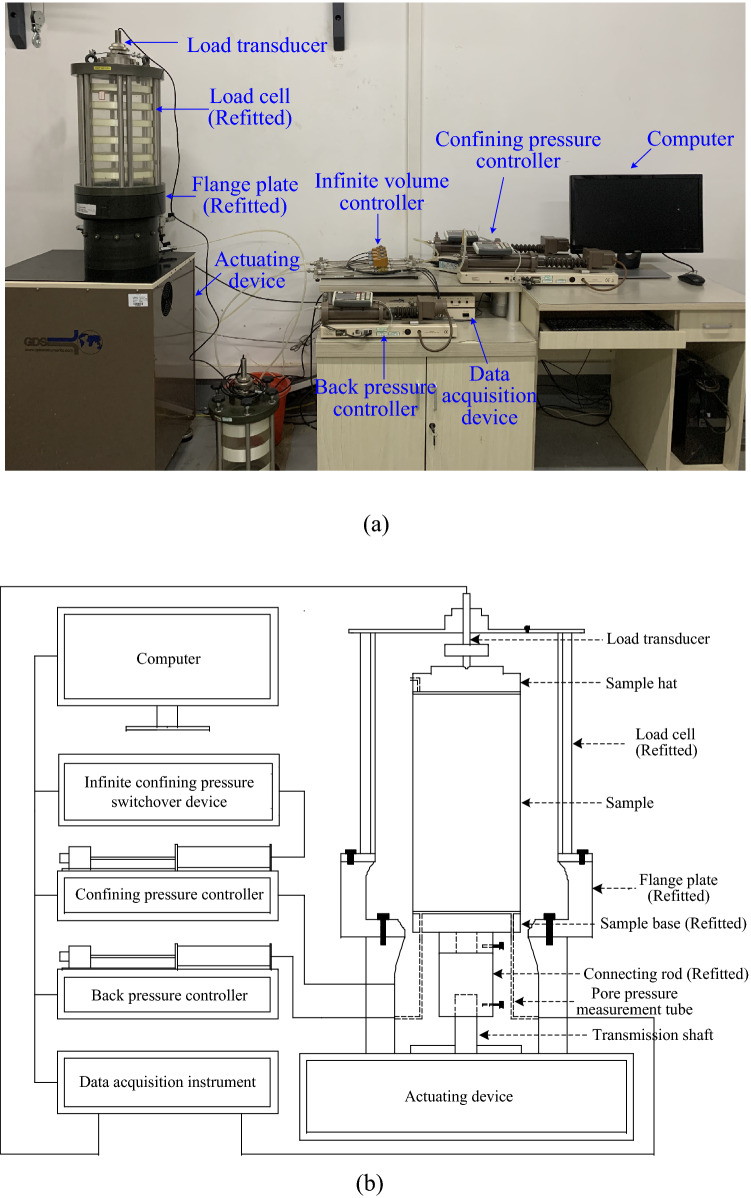
GDS triaxial test apparatus in (**a**) photo; and (**b**) diagram.

The triaxial test apparatus used in this study was designed and manufactured by GDS Instruments, Ltd, as shown in Fig. 3. The UGM samples were placed at the center of load cell initially. The confining pressure controller, which can provide the largest confining pressure of 2 MPa, is connected with triaxial cell with plastic tube to compel de-aired water into the cell to impose confining pressure. The actuating device at the bottom is motivated in the loading process to apply axial cyclic stress to the gravel sample. At the same time, load and displacement transducers can collect data in real time automatically. For the UGM with a given DoC, both monotonic and cyclic triaxial tests were performed under the confining pressure of 40 kPa and 60 kPa. The monotonic triaxial test was performed to obtain the peak strength of the UGM samples that can provide guidance for the loading scheme of cyclic triaxial tests. Eight samples were involved in this test program totally, with four samples with DoC of 0.95 and another four with DoC of 1.0, as can be seen in Table 2.

**Table 2:** Test scheme.

Sample no	Degree of compaction	Confining pressure (kPa)	Test type
I	0.95	40	Monotonic test
II	0.95	40	Cyclic test
III	0.95	60	Monotonic test
IV	0.95	60	Cyclic test
V	1.00	40	Monotonic test
VI	1.00	40	Cyclic test
VII	1.00	60	Monotonic test
VIII	1.00	60	Cyclic test

The test was conducted in the drained condition. Moreover, shearing velocity was set as low as 0.01 mm/min to minimize the pore pressure in the loading process. The shearing was ceased once the axial strain of the samples reached to 10%. After the monotonic tests, the peak shear strength (*σ*_1_ − *σ*_3_)_*f*_ can be obtained for the UGM samples.

**Figure 4:**
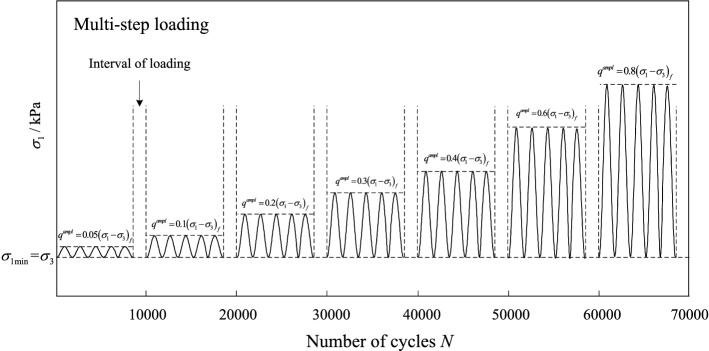
The cyclic loading program.

The cyclic triaxial test was also performed with the drainage valve opening in the loading process. A multi-step loading program was adopted in this study with seven stages, as shown in Fig. 4. Since UGM were usually adopted to construct the subgrade and buried relatively shallow, the confining pressures of 40 kPa and 60 kPa were applied to conduct the cyclic test. Similarly, the UGM samples with DoC of 0.95 and 1.00 were prepared for the cyclic test. Since samples with different DoC presented different peak shear strengths (*σ*_1_ − *σ*_3_)_*f*_ under different confining pressures, the cyclic stress *q*^*ampl*^ was applied for each sample in accordance with (*σ*_1_ − *σ*_3_)_*f*_, as shown in the following equation:

1 $$q^{ampl} = CSR \cdot \left( {\sigma_{1} - \sigma_{3} } \right)_{f}$$

where *CSR* is defined as cyclic stress ratio, which is the ratio of the cyclic stress to the static peak strength. *σ*_1_and *σ*_3_ represent the major and minor principal stress, and (*σ*_1_ − *σ*_3_) denotes the deviatoric stress applied in the shear stage. For each sample, *CSR* equals to 5%, 10%, 20%, 30%, 40%, 60%, 80% respectively in the seven stages of the multi-step loading program. The UGM samples were loaded for 10,000 cycles in every loading stage at a frequency of 1 Hz. The test data was recorded at 50 points for every loading cycle.

Before normal cyclic loading, a slow loading at a frequency of 0.01 Hz was firstly applied to the sample to clear off the contact irregularities at the top of the sample. The interval between two loading stages should be set long enough to allow the dissipation of pore water pressure in case that it will affect the next loading stage. Several loading amplitudes were allowed for a UGM sample in the multi-step loading program, thus the influences of sample variability and number were reduced.

## Test results

### Peak shear strength

**Figure 5:**
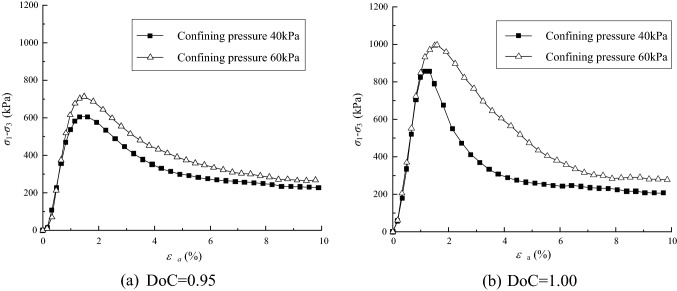
The relationship between deviatoric stress and axial strain for samples with DoC of (1) 0.95 and (2) 1.00.

The deviatoric stress was plotted against axial strain for the UGM samples with DoC of 0.95 and 1.00, as shown in Fig. 5. It can be seen that the deviatoric stress *σ*_1_ − *σ*_3_ of the UGM samples increases with axial strain *ε*_*a*_ initially, but it decreases with the increase of *ε*_*a*_ when *ε*_*a*_ > 1.5% until *σ*_1_ − *σ*_3_ levels off to its residual value. The investigated densely-compacted UGM samples demonstrate strain softening features under the confining pressures of 40 kPa and 60 kPa.

Furthermore, the peak shear strength (*σ*_1_ − *σ*_3_)_*f*_ of the UGM sample with a given DoC increases significantly with the confining pressure. When the confining pressure increases from 40 to 60 kPa, the peak strength of the sample with DoC = 0.95 rises from 605.5 to 713.4 kPa, and the sample with DoC = 1.00 presents peak strength that increases from 855.9 to 955.5 kPa. Besides, all of the samples reach to the peak strengths on the condition that the axial strain *ε*_*a*_ approximately arrives at 1.5%.

### Permanent deformation

**Figure 6:**
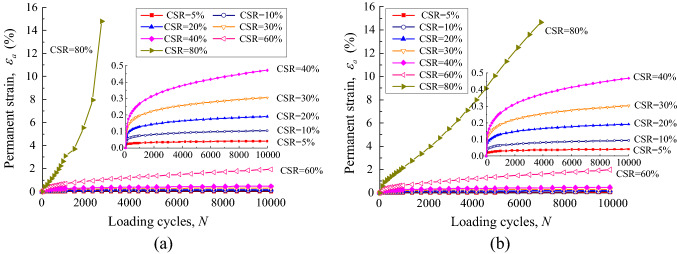
Permanent deformation plotted against loading cycles for UGM samples with DoC = 0.95 at the confining pressure of (**a**) 40 kPa and (**b**) 60 kPa.

During the cyclic loading process, both pore pressure and axial deformation were monitored in real time to record the long-term properties of UGM samples. Due to the drained condition and low loading frequency, pore water pressure stayed all along below 2 kPa, indicating that the influence of pore water can be neglected. The UGM samples present quasi-sine-shaped deformation curves due to the sine-shaped cyclic stress. Both elastic and permanent deformation existed in each loading cycle. The elastic deformation was recovered time after time in tens of thousands of load times, while the permanent deformation is accumulated gradually. The permanent strain can be plotted against loading cycles to illustrate the long-term deformation properties of the UGM samples. Figure 6 is the permanent strain curve for the sample with DoC = 0.95 under the confining pressures of 40 kPa and 60 kPa.

As depicted by Fig. 6a, the permanent strain *ε*_*a*_ of the sample developed sluggishly and finally ceased at a relatively low value on the condition that CSR stayed less than 30%. When CSR increased to more than 30%, a significant increasing trend of *ε*_*a*_ can still be observed at the end of each loading stage, indicating that *ε*_*a*_ was likely to develop continuously as long as cyclic loading was applied. As CSR increased to 80%, not only *ε*_*a*_ increased with loading cycles *N*, but also the growth rate of the permanent strain also increased continuously. The UGM sample collapsed rapidly when loading cycles increased to less than 3000. Therefore, the UGM sample demonstrated quite different permanent deformation properties at high stress levels compared with those at low stress levels. Figure 6b depicts the permanent strain *ε*_*a*_ of the UGM sample with DoC = 0.95 at the confining pressure of 60 kPa. It was not difficult to find that the sample with DoC = 0.95 presented quite similar deformation properties at confining pressure of 60 kPa to those at confining pressure of 40 kPa.

**Figure 7:**
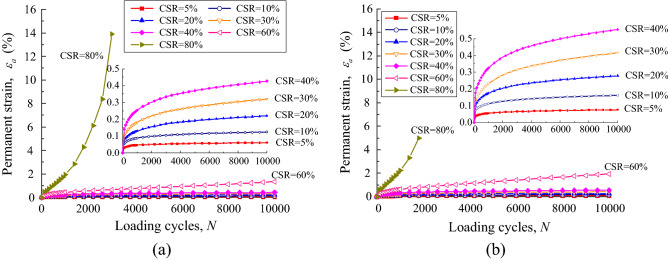
Permanent deformation plotted against loading cycles for gravel samples with DoC = 1.00 at the confining pressure of (**a**) 40 kPa and (**b**) 60 kPa.

The relationship between permanent strain *ε*_*a*_ and loading cycles for sample with DoC = 1.00 is demonstrated in Fig. 7. Similarly, the strain of the sample tended to become stable rapidly at CSR = 5% under both confining pressures, and furthermore *ε*_*a*_ was also able to converge to a certain value and remained steady with subsequent loading cycles on the condition of CSR < 30%. As CSR increased beyond 30%, *ε*_*a*_ developed continuously with loading cycles, leading to the specimen breaking down sooner or later. Moreover, the growth rate of *ε*_*a*_ also increased with loading cycle on the condition of CSR = 80%, which implied that *ε*_*a*_ developed in an accelerated manner and the sample collapsed momentarily. By investigating the permanent strain properties of samples with different DoC under different confining pressures, it was remarkable that the investigated UGM samples demonstrated similar long-term deformation properties as the permanent strain *ε*_*a*_ was plotted against loading cycles *N* under different CSR*s*, regardless of degree of compaction and confining pressure *σ*_3_.

## Strain development analysis

### Permanent strain rate analysis

**Figure 8:**
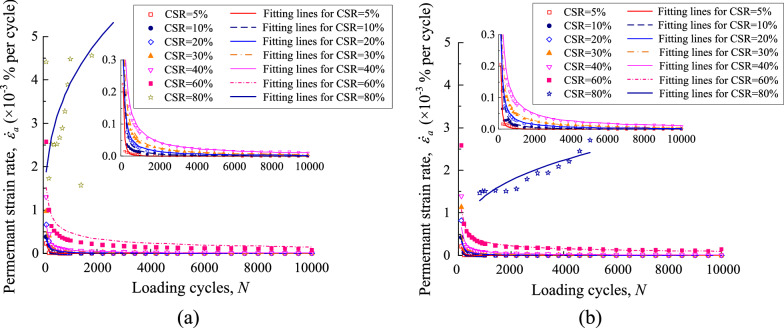
Permanent strain rate against loading cycles for gravel samples with DoC = 0.95 at the confining pressure of (**a**) 40 kPa and (**b**) 60 kPa.

**Figure 9:**
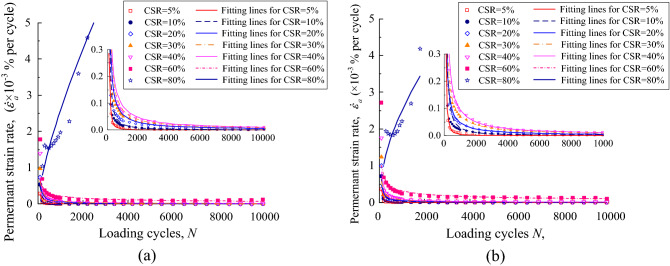
Permanent deformation rate against loading cycles for gravel samples with DoC = 1.00 at the confining pressure of (**a**) 40 kPa and (**b**) 60 kPa.

The permanent strain rate of the UGM samples was calculated in accordance with the permanent strain properties for every loading stage. The permanent strain rate against loading cycles was plotted in Figs. 8 and 9 for the UGM samples with different DoC. Figure 8a demonstrates the relationship between the permanent strain rate $\dot{\varepsilon }_{a}$ and loading cycles *N* for the samples with DoC = 0.95 under the confining pressure of 40 kPa. The scattered points represented the strain rate $\dot{\varepsilon }_{a}$ that is calculated from the measured strain. As presented, $\dot{\varepsilon }_{a}$ showed quite different development properties under different CSRs for a given sample. On the condition of CSR = 5%, $\dot{\varepsilon }_{a}$ decreased sharply in the first 1000 loading cycles and returned almost to zero before the 2000-th loading cycle, which corresponded to the permanent strain curve that converged rapidly in the first 2000 loading cycles. As CSR increased to 10% and 20%, the attenuation of $\dot{\varepsilon }_{a}$ against loading cycles was weakened, even although $\dot{\varepsilon }_{a}$ also decreased almost to zero finally. When CSR developed to 30%, 40% and 60%, the strain rate $\dot{\varepsilon }_{a}$ never decreased to zero despite the fact that it fell off all along with the loading cycles. This implied that the permanent strain will always develop as long as cyclic loading was applied. Finally, $\dot{\varepsilon }_{a}$ even increased with loading cycles on the condition of CSR = 0.8, indicating that the permanent strain increased sharply until the sample collapsed. Thus, it can be seen that strain rate $\dot{\varepsilon }_{a}$ presented a close relationship with the loading cycles *N*, and furthermore this relationship was quantitatively determined by CSR applied on the UGM sample.

**Figure 10:**
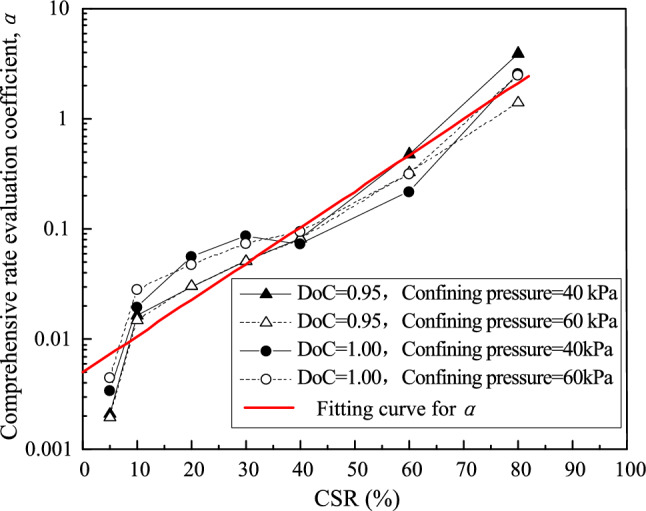
The relationship between comprehensive evaluation coefficient *α* and CSR.

The permanent strain rate $\dot{\varepsilon }_{a}$ of the DoC = 0.95 sample under the confining pressure of 60 kPa was presented in Fig. 9b. It was interesting to find that the strain rate $\dot{\varepsilon }_{a}$ presented quite similar development properties on the condition of *σ*_3_ = 60 kPa to those on the condition of *σ*_3_ = 40 kPa. Furthermore, the samples with DoC = 1.00 also presented similar evolvement properties of strain rate to the samples with DoC = 0.95, as shown in Fig. 10. In other words, the strain rate $\dot{\varepsilon }_{a}$ of the UGM samples presented a solid relationship against loading cycles *N* under the effect of different CSRs, regardless of the DoC and confining pressure.

A power law function with a negative exponent was introduced to illustrate the correlation of permanent strain rate $\dot{\varepsilon }_{a}$ with loading cycles *N*, as shown by the following equation:

2 $$\dot{\varepsilon }_{a} = \alpha N^{ - \beta }$$

where *α* and *β* are constants that varied with CSRs for a given sample under a given confining pressure. The parameter *α* refers to the average strain rate for a given amplitude of cyclic load. The larger average level of strain rate at different loading stages, the higher value of *α*. The parameter *α* can be termed as comprehensive evaluation coefficient for permanent strain rate. Furthermore, the parameter *β* is an indicator to describe the successional trend for strain rate curve. As demonstrated by the equation, the strain rate $\dot{\varepsilon }_{a}$ increased with loading cycles *N* on the condition of *β* < 0, indicating that the sample will collapse in limited loading cycles. As *β* becomes positive, strain rate $\dot{\varepsilon }_{a}$ decreased with loading cycles *N*, but permanent strain *ε*_*a*_ increased against *N*. The final state of UGM sample is ambiguous. To clarify the final state of UGM sample, the permanent strain can be expressed by the following equation integrated from Eq. (3):

3 $$\varepsilon_{a} = \left\{ {\begin{array}{ll} {\frac{\alpha }{1 - \beta }N^{1 - \beta } + \varepsilon_{a,\max } ,} & \quad {{\kern 1pt} \beta > 1} \\ {\alpha \ln N,} & \quad {{\kern 1pt} \beta = 1} \\ {\frac{\alpha }{1 - \beta }N^{1 - \beta } ,} & \quad {{\kern 1pt} \beta < 1} \\ \end{array} } \right.$$

where *ε*_*a*,max_ is the maximum axial strain with loading cycles *N* becoming infinity on the conditions of low cyclic amplitude level and *β* > 1. As *β* stays greater than unity under low cyclic amplitudes, the expression *αN*^(1−*β*)^/(1 − *β*) is negative. Moreover, the absolute value of *αN*^(1−*β*)^/(1 − *β*) advances gradually towards zero as *N* increases to infinity, indicating that *ε*_*a*_ of the UGM sample will finally converge to *ε*_*a*,max_ and the sample will remain steady. As *β* ≤ 1, permanent strain *ε*_*a*_ of the UGM sample increases continuously with *N*, and the sample will finally collapse. Thus, *β* can be defined as developing tendency coefficient that can depict the strain rate developing characteristics quantitatively.

The relationship between strain rate $\dot{\varepsilon }_{a}$ and loading cycles *N* of the investigated samples was fitted by Eq. (3). The fitting parameters *α* and *β*, together with correlation coefficients, were demonstrated in Table 3. The fitting strain curves were also presented in Figs. 8 and 9. All of the fitting curves have relatively high correlation coefficients that are more than 0.6, averaging at 0.78 and 0.75 for the samples with DoC = 0.95 and DoC = 1.00 respectively. Furthermore, as shown in Figs. 8 and 9, it was obvious that the fitting curves matched well with the measured strain rate, indicating that Eq. (3) can be adopted to illustrate the development properties of strain rate for the UGM samples.

**Table 3:** Fitting parameters for the UGM samples under different CSRs.

DoC	*σ*_3_/kPa	CSR	*α*	*β*
0.95	40	5	0.002	2.01
		10	0.017	1.37
		20	0.030	1.04
		30	0.051	1.00
		40	0.083	0.90
		60	0.478	0.50
		80	3.922	− 0.32
0.95	60	5	0.002	2.01
		10	0.015	1.41
		20	0.030	1.04
		30	0.051	0.97
		40	0.082	0.88
		60	0.326	0.52
		80	1.396	− 0.34
1.00	40	5	0.003	1.91
		10	0.020	1.39
		20	0.056	1.00
		30	0.087	0.93
		40	0.074	0.94
		60	0.218	0.48
		80	2.581	− 0.73
1.00	60	5	0.004	1.89
		10	0.028	1.35
		20	0.048	0.99
		30	0.074	0.89
		40	0.095	0.94
		60	0.317	0.50
		80	2.501	− 0.54

### Development tendency analysis

The comprehensive evaluation coefficient *α* and developing tendency coefficient *β* can be obtained by fitting the measured strain rate of the UGM samples under different CSRs. The coefficients were plotted against CSR to analyze the developing tendency of permanent strain rate in different loading stages. It is found that *α* increased exponentially with CSR. Therefore, the logarithm of *α* was correlated with CSR for the tested UGM samples, as shown in Fig. 10. *α* demonstrated quite similar relationships with CSR for the UGM samples with different DoC and confining pressures. Moreover, there was an obvious linear relationship between the logarithm of *α* and CSR. A regression analysis was performed to give the following equation a correlation coefficient as high as 0.91:

4 $$\log_{10} \alpha = 3.28CSR - 2.30$$

where log_10_ is a base-10 logarithm. Furthermore, the comprehensive evaluation coefficient *α* can be evaluated from the following equation with CSR known:

5 $$\alpha = 10^{3.28CSR - 2.30}$$

Since the samples with different DoC and confining pressures *σ*_3_ presented similar development properties of *α* against CSR, the relationship between *α* and CSR is insusceptible to DoC and *σ*_3_. With this equation, the comprehensive evaluation coefficient *α* can be calculated to evaluate the average strain rate for a given CSR.

**Figure 11:**
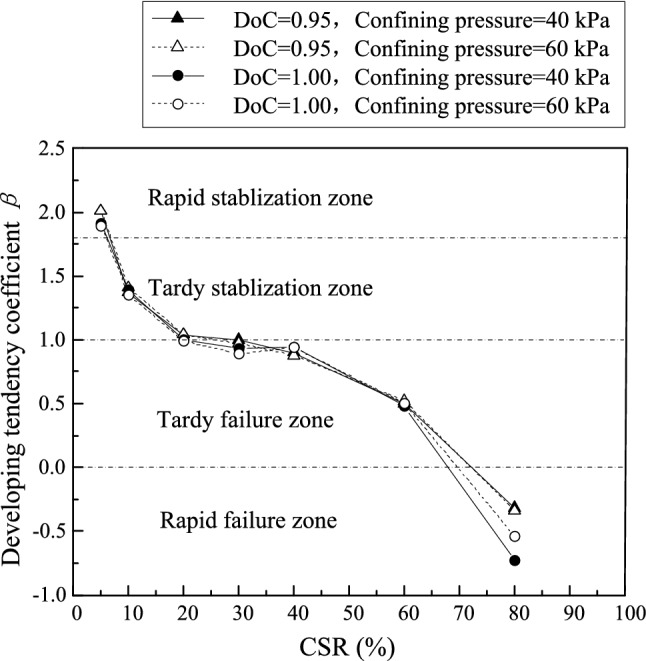
The relationship between development tendency coefficient *β* and CSR.

More importantly, the correlation of developing tendency coefficient *β* with CSR is a more critical relationship that determines evolvement characteristics of permanent strain rate against loading cycles. As shown in Fig. 11, samples with different DoC presented a normalized curve for developing tendency coefficient *β* under different confining pressures. This also implied that the developing tendency of permanent strain rate is determined by CSR separately and insusceptible to DoC and confining pressure.

On the condition of CSR = 5%, the developing tendency coefficient *β* remained more than 1.8 for the investigated UGM samples. In this circumstance, the strain rate converged to zero rapidly in the first 2000 loading cycles, and the permanent strain leveled off to a relatively low value. It can be deduced that the sample could stay steady all along despite of infinite loading cycles. Therefore, this status can be defined as “rapid stablization” on the condition of *β* ≥ 1.8. When 1.0 ≤ *β* < 1.8, CSR equaled to 10% and 20%, and the strain rate finally converged to zero in 10,000 loading cycles. Although more loading cycles were needed to make the permanent strain steady compared with the rapid stabilization status, the strain could also level off finally. The permanent strain development features corresponding to 1.0 ≤ *β* < 1.8 can be termed as “tardy stablization”. As CSR increased to 30%, 40% and 60%, *β* located between 0 and 1. In this situation, the strain rate of the investigated UGM samples decreased with loading cycles, while permanent strain developed continuously and never converged. Meanwhile, the development features of both strain rate and permanent strain were also evidenced by the Eqs. (2) and (3), which were verified to illustrate the long-term deformation properties of UGM samples perfectly. Thus, the sample will finally break down due to the ever-increasing permanent strain, and this status can be referred to as “tardy failure”. Once CSR increased to 80%, *β* fell below 0 immediately. This implied that the strain rate rose with loading cycles, and the sample collapsed in limited time, which corresponded to “rapid failure”.

As discussed above, comprehensive evaluation coefficient *α* depicts the average permanent strain rate for a given loading stage, while developing tendency coefficient *β* demonstrates the evolvement tendency of strain rate against loading cycles. As a coefficient that is insusceptible to DoC and confining pressure, *β* determines the long-term deformation properties of UGM samples directly. Correspondingly, the subgrade in high-speed railway, which is constructed mainly by UGM, should be designed by taking developing tendency coefficient *β* into consideration. For example, since unballasted track raised a more rigorous requirement for permanent deformation to the substructure, substructure fillings should be controlled to fall into the rapid stabilization zone, which implies that *β* should be more than 1.8. Moreover, ballasted track should have subgrade fillings that stays in tardy stabilization zone and *β* should be set between 1.0 and 1.8, since the limited deformation can be supplemented by replenishing new ballast. As for the branch railway that requires a short service life, the fillings can be controlled in tardy failure zone (0 ≤ *β* < 1.0), which is economical and practical in engineering practice. Therefore, *β* is a critical parameter that determines the long-term deformation tendencies of UGM-constructed soil structure.

## Numerical simulations

### Axial strain by DEM

To reveal the strain development mechanism of the triaxial UGM samples and verify the reasonability of long-term deformation analysis presented above, discrete element method (DEM) was adopted to identify the long-term deformation properties of UGM samples under different CSRs. In the numerical modeling, the PFC 2D software was employed to perform the biaxial compression tests for the UGM samples. To prepare the simulated UGM samples, the images of real gravel particles were firstly dealt with binary conversion to receive the two-dimensional outline. Then, the circle elements that are partially overlapped with each other were created within the closed outline to generate the clump model of each UGM particle. In this way, the contact force between clump models can be captured by calculating the contact force between circle elements, which could improve the computational efficiency with irregular particle shape taken into consideration.

Furthermore, a flexible membrane was adopted to enclose the coarse particles to generate the UGM samples with the diameter of 150 mm and height of 300 mm. Some micro parameters adopted are listed in Table 4. The UGM samples used in this study were in the same batch with those adopted in the previous study by Liu et al., the density of the UGM samples can be set as 2740 kg/m^3^^25^. The porosity was determined by DEM-simulated vibration compaction test in a cylinder container with diameter of 150 mm and height of 300 mm. Results show that the densest UGM sample has the porosity of 0.095 at the given grading characteristics. Thus, the value of porosity was set to be 0.1 in this simulation test to represent the UGM sample with DoC of 0.95. Effective modulus, normal shear stiffness ratio, interparticle friction coefficient, particle local damping coefficient and anti-rolling friction coefficient (particle size 1.6–2 mm) were determined complying with the previous studies^26–31^. The connection between flexible membrane and coarse particles is analyzed in terms of the linear parallel bond model, the relevant parameters were set in accordance with Shi et al.^32^.

**Table 4:** Some micro parameters adopted in DEM analysis.

Parameters	Value
Density	2740 kg/m^3^
Porosity	0.1
Effective modulus	3 × 10^9^ N/m^2^
Normal shear stiffness ratio	1.0
Interparticle friction coefficient	0.7
Particle local damping coefficient	0.7
Particle anti-rolling friction coefficient (particle size1.6–2 mm)	3.5
Tensile strength coefficient of flexible film (parallel bond)	1 × 10^300^
Cohesion coefficient of flexible film (parallel bond)	1 × 10^300^

**Figure 12:**
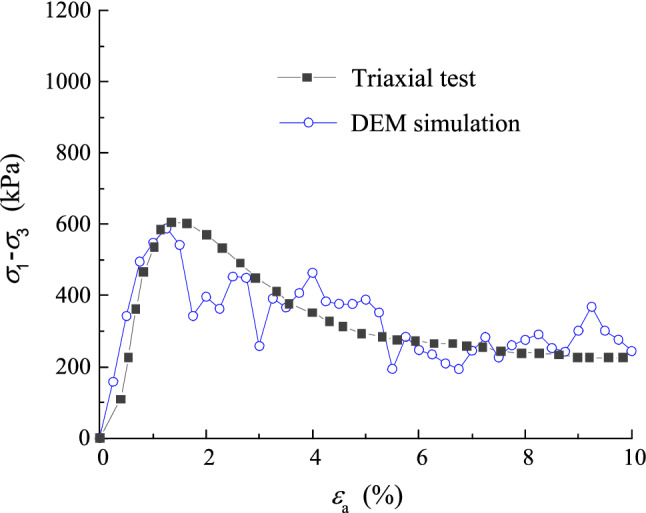
Comparison of DEM-simulated stress–strain curve with the measured one in triaxial test.

Both monotonic and cyclic tests were performed on the simulated UGM samples under the servo (confining) pressure of 40 kPa. The deviatoric stress of the simulated sample was plotted against axial strain to compare with the stress–strain curve of the triaxial sample with DoC of 0.95, as shown in Fig. 12. It was found that the peak shear strength of the sample in DEM test was approximately 600 kPa, which was rather close to measured one in triaxial test. Besides, the correlation of deviatoric stress with axial strain calculated from DEM simulation almost developed along the stress–strain curve of the triaxial sample. It should be noted that the fluctuation in the DEM-calculated stress–strain curve can be attributed to the fact that the size of finest particles in the simulated sample were remarkably greater than those in the triaxial sample. Actually, the essence of the finest particle in DEM sample is an equivalent representative of partial fine-grained particles of the UGMs. Therefore, the established DEM sample can be adopted to simulate the UGMs reported in this study.

**Figure 13:**
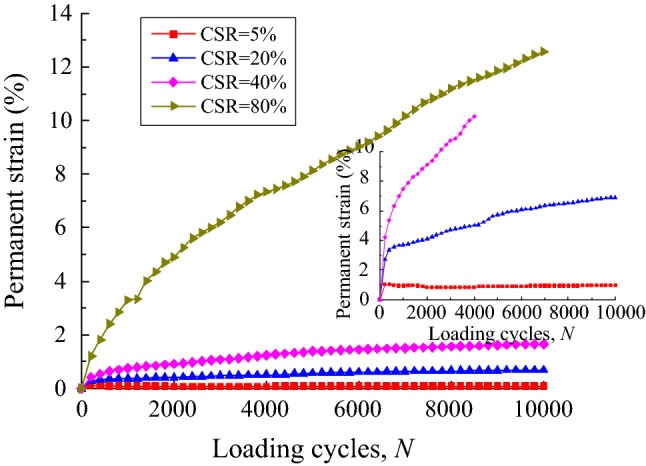
DEM-calculated permanent strain plotted against loading cycles for UGM samples at different CSRs.

**Figure 14:**
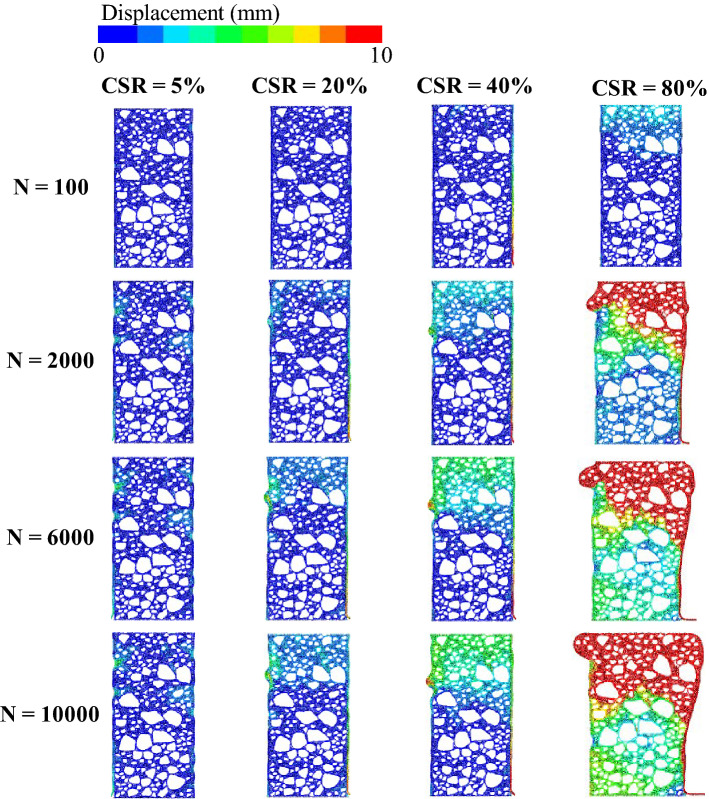
Axial strain of the simulated UGM samples under different CSRs obtained by discrete element analysis.

The cyclic stress was applied on the UGM sample in accordance with Eq. (1), which is similar to the loading scheme in the triaxial test. The DEM-calculated permanent strain of the samples was plotted against loading cycles on the condition of CSR = 5%, 20%, 40% and 80% respectively, as shown in Fig. 13. It is obvious that the permanent strain under these four CSRs demonstrate quite different developing tendencies that bring into correspondence with those in triaxial test, although definite strain value obtained by DEM is slightly higher than that measured in triaxial test under each CSR level. Furthermore, the local deformation characteristics of UGM samples under different CSRs was recorded at loading cycles of 100, 2000, 6000 and 100,000, as shown in Fig. 14.

As presented, the displacement at the top of the sample stayed at a very low level when CSR equals to 5%, 20% and 40% respectively on the condition of loading cycles *N* = 100, indicating that the axial strain of the sample can be neglected under the three CSR levels. The CSR = 80% sample showed relatively remarkable displacement of 2–3 mm at the top of the specimen under the effect of 100 loading cycles, which corresponded to axial strain of 0.67–1%. When the loading cycles increased to 2000, the CSR = 80% sample showed great vertical displacement that was larger than 10 mm at the upper end of the specimen, which was quite different from the slight displacement demonstrated in the other three samples with CSR = 5%, 20% and 40%. Moreover, the axial strain of the CSR = 40% sample reached 1% on the condition of *N* = 2000, which was slightly greater than those of the samples with CSR = 5% and 20%.

With the loading cycles of 6000, serious deformation and remarkable axial strain occurred for the CSR = 80% and CSR = 40% sample respectively, and the upper portion of the specimen with CSR = 20% presented vertical displacement that was higher than the CSR = 5% sample. However, both CSR = 5% and CSR = 20% samples had axial strains that were significantly less than those of CSR = 40% and CSR = 80% sample. As loading cycles increased to 10,000, even though the CSR = 20% sample accumulated greater axial strain compared with the CSR = 5% sample as the loading cycle increases, axial strains of both samples stayed at a relatively low level all along. More importantly, axial strains of both samples increased gently when loading cycles increased from 6000 to 10,000. On the other hand, great strains were accumulated for the CSR = 40% and CSR = 80% samples. Furthermore, the permanent strains of both samples increased continuously with the loading cycles. Above all, local deformation properties of the UGM samples presented great variability under different cyclic stress levels, which further resulted in different deformation developing tendencies.

### Interparticle force chain analysis

**Figure 15:**
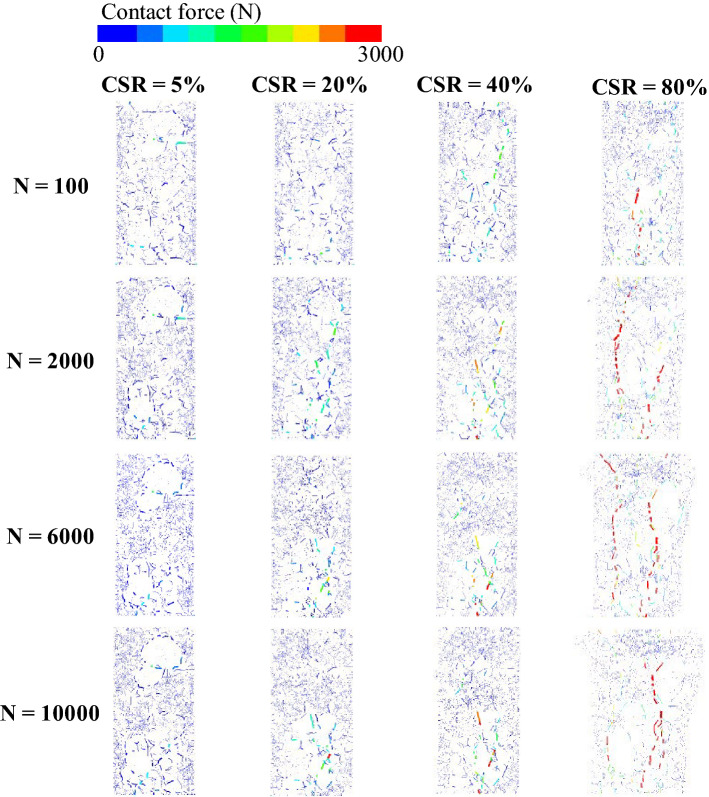
Interparticle force chain characteristics of the simulated UGM samples under different CSRs obtained by discrete element analysis.

To further clarify the long-term deformation mechanism of UGM samples under different CSRs, the interparticle force chain characteristics were collected at loading cycles *N* = 100, 2000, 6000 and 10,000 respectively, as shown in Fig. 15. It can be seen that all of the interparticle forces stayed at a relatively low level for the CSR = 5% and CSR = 20% samples on the condition of *N* = 100. A small number of moderate and strong force chains were observed in the CSR = 40% and CSR = 80% samples respectively, and most force chains in these two samples still hold at a relatively low level. As the loading cycles increased, a mass of strong force chains generated and sustained throughout the whole sample height for the sample with CSR = 80%, which led to the rapidly increasing permanent strain. Therefore, this sample with CSR = 80% corresponded to rapid failure. However, a series of moderate force chains existed in the CSR = 40% sample at loading cycles *N* = 2000. Moreover, slight strong force chains emerged at the middle of the sample and gradually moved downwards with the increased loading cycles. When the loading cycles reached to 10,000, a significant number of moderate and strong chains generated from the middle to the bottom for the samples with CSR = 40%, corresponding to tardy failure state.

As for the sample with CSR = 5%, moderate and strong force chains will never generate in the whole loading process till *N* = 10,000. This implied that the permanent strain of the UGM sample reached to rapid stabilization status in just a few loading cycles. However, despite the fact that no moderate or strong force chains were formed for the CSR = 20% sample on the condition of *N* = 100, a small amount of moderate force chains appeared as the loading cycles increased to more than 2000. Only a short strong force chain generated at the sample bottom when *N* reached to 10,000, resulting in axial permanent strain that converged to a certain value with increased loading cycles.

**Figure. 16:**
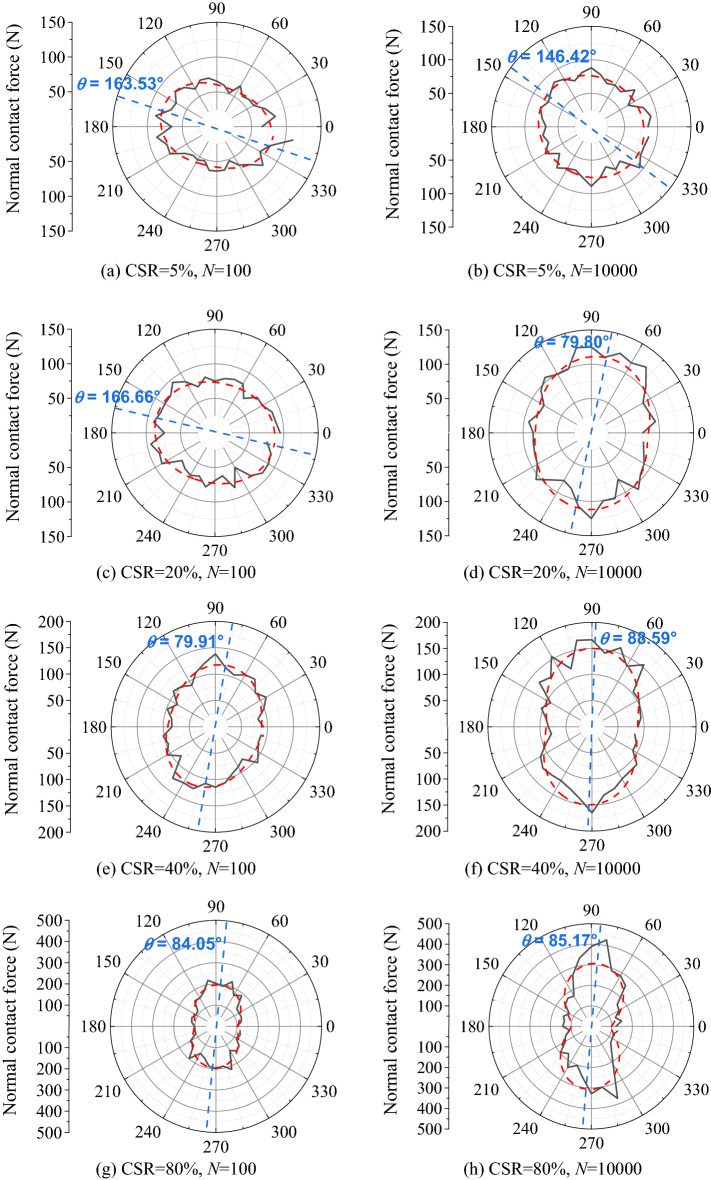
Distribution features of normal contact force under different CSRs.

To quantitatively illustrate the development properties of interparticle contact forces under different CSRs, the distribution features of normal contact forces were analyzed for the DEM sample in both initial loading state (*N* = 100) and final one (*N* = 10,000), as shown by the black solid lines in Fig. 16. Furthermore, trigonometric function was adopted to fit the distribution of contact forces to calculate principal stress direction angle *θ*. The fitting trigonometric curves was presented by red dash lines in Fig. 16. It is presented in Fig. 16 that:

- As CSR = 5%, normal contact forces tended to present horizontal distribution on the condition of *N* = 100, leading to the maximum contact force of 111.03 N and *θ* = 163.53°. As loading cycles increased to 10,000, normal contact forces still distributed horizontally in spite of the tendency of vertical development. The maximum contact force was 95.25 N, and the principal stress direction angle *θ* was 146.42°. This corresponded to the annular force chain state presented in Fig. 15, indicating that the interparticle contact states of the sample remained steady during the cyclic stress period.
- As CSR = 20%, normal contact forces also demonstrated approximately horizontal direction at *N* = 100. The maximum contact force was 95.38 N, corresponding to principal stress direction angle *θ* of 166.66°. However, normal contact force converted to distribute vertically when *N* arrived at 10,000, resulting in the maximum contact force of 124.98 N and *θ* = 79.80°. This implied that the interparticle contact state remained steady initially, but gradually converted to vertical distribution to resist deformation with loading cycles increasing. Nevertheless, the maximum contact force varied slightly during the whole loading process, which indicated the sample will stay steady.
- As CSR = 40%, normal contact forces presented vertical distribution initially on the condition of *N* = 100. The maximum contact force was 138.36 N, and principal stress direction angle *θ* was 79.91°. As *N* increased to 10,000, normal contact forces still distributed vertically, leading to the maximum contact force of 168.13 N and *θ* = 88.59°. It was presented that normal contact forces acted as a role to resist deformation in the initial loading stage, but the magnitude of maximum contact force was not large enough to lead to sample collapse initially. However, the magnitude of the maximum contact force increased continuously with loading cycles, which will finally induce failure under the effect of cyclic loading.
- As CSR = 80%, normal contact forces distributed vertically in both initial and final loading stages. *θ* varied from 84.05° to 85.17°, and the maximum contact force increased from 223.91 N to 427.47 N. This implied that the UGM sample had a necessity to resist deformation initially. Moreover, the maximum contact force at *N* = 10,000 was almost twice the force at *N* = 100, indicating that the contact force rose sharply with loading cycles on the condition of CSR = 80%. This will undoubtedly result in sample collapsed in limited loading cycles.

With the discussion presented above, the interparticle force chain characteristics induced by different levels of cyclic stress varied significantly for the UGM samples, which may be the origin of different long-term deformation properties. As CSR increased from 5 to 80%, the amount of moderate and strong force chains gradually increased to a relatively high level. Correspondingly, different degrees of increase for the maximum contact force and varying principal direction angle were presented for the simulated samples with different CSRs, which may lead to the permanent strain evolving from rapid stabilization to tardy stabilization, then to tardy failure, and finally to rapid failure. The DEM simulations validated the proposed classification criterion by the power function fitting technique for the permanent deformation properties of UGM materials.

## Conclusions

Medium-sized cyclic triaxial tests were performed on unbound granular material (UGM) under different confining pressures to investigate the long-term deformation properties. Discrete element method (DEM) was adopted to conduct biaxial compression test for simulated samples to illustrate the deformation mechanisms. Some significative conclusions can be drawn as follows:

1. The long-term deformation characteristics of UGM samples vary significantly in terms of cyclic stress levels. As cyclic stress ratio (CSR) increases from 5 to 80%, the UGM samples can transform from stabilization to failure state. Both states can be subdivided into rapid and tardy one in accordance with CSR for the UGM samples, regardless of degrees of compaction and confining pressures.
2. A power law function with a negative exponent can be adopted to analyze the relationship between permanent strain rate of UGM sample and loading cycles, and the exponent *β* is a deformation tendency indicator that can be selected to analyze the long-term properties quantitatively. As *β* > 1.8, the UGM sample falls into rapid stabilization zone with a given CSR. The deformation developing tendency correspond to tardy stabilization and tardy failure on the condition of 1.0 ≤ *β* < 1.8 and 0 ≤ *β* < 1.0. As *β* decreases to less than 0, the sample will collapse rapidly in a few moments.
3. The proposed classification criterion in this study has been validated by DEM analysis with PFC 2D. Results show that variation of CSR results in different interparticle force chain characteristics, which may be the origin of different long-term deformation properties of UGM materials. Weak force chains and horizontal principal direction angle at CSR = 5% and 20% lead to steady permanent strains in limited loading cycles. Moderate to strong force chains and vertical principal direction angle at high CSR levels produce ever-increasing deformation that finally leads to sample collapse.

## Supplementary Information

Supplementary Tables.

## Data Availability

All data generated or analysed during this study are included in this published article (and its supplementary information files).
