# Supplementary material for: Characterization of permanent deformation properties of densely-compacted unbound granular materials subjected to cyclic loading
Source: Sci Rep. 2023 Mar 6;13:3731. doi: 10.1038/s41598-023-30635-7 (PMC9988872; doi:10.1038/s41598-023-30635-7)
Supplement: Supplementary file 1 — Supplementary Tables. [file 41598_2023_30635_MOESM1_ESM.docx]

Table 1 Grain size distribution for the UGM in this study

| Particle size / mm | Percent passing by weight / % |
| --- | --- |
| 31.5 | 100 |
| 19 | 82 |
| 9.5 | 66 |
| 4.75 | 52 |
| 2.36 | 40 |
| 0.5 | 22 |
| 0.25 | 14 |
| 0.075 | 3 |

Table 2 The relationship between deviatoric stress and axial strain for the UGM samples

| CD=0.95 | | | | CD=1.00 | | | |
| --- | --- | --- | --- | --- | --- | --- | --- |
| *σ*_3_=40 kPa | | *σ*_3_=60 kPa | | *σ*_3_=40 kPa | | *σ*_3_=60 kPa | |
| *ε_a_*/% | *σ*_1_-*σ*_3_/kPa | *ε_a_*/% | *σ*_1_-*σ*_3_/kPa | *ε_a_*/% | *σ*_1_-*σ*_3_/kPa | *ε_a_*/% | *σ*_1_-*σ*_3_/kPa |
| 0 | 0 | 0 | 0 | 0 | 0 | 0 | 0 |
| 0.1655 | 14.7647 | 0.1655 | 9.1538 | 0.1645 | 58.184 | 0.1656 | 61.8899 |
| 0.3312 | 108.9103 | 0.3312 | 70.1134 | 0.329 | 179.6977 | 0.3312 | 205.0052 |
| 0.4967 | 227.4091 | 0.4967 | 212.9396 | 0.4935 | 335.1924 | 0.4967 | 369.795 |
| 0.6623 | 357.2689 | 0.6623 | 374.4137 | 0.658 | 521.265 | 0.6623 | 552.0057 |
| 0.8279 | 469.6445 | 0.8279 | 518.1036 | 0.8225 | 705.5786 | 0.8279 | 721.9982 |
| 0.9935 | 536.8222 | 0.9935 | 615.0478 | 0.987 | 823.8911 | 0.9935 | 849.3953 |
| 1.1591 | 581.2113 | 1.1591 | 675.8338 | 1.1515 | 855.0004 | 1.1591 | 930.987 |
| 1.3247 | 605.2488 | 1.3247 | 704.0145 | 1.316 | 855.9321 | 1.3247 | 970.7775 |
| 1.6024 | 605.4716 | 1.4903 | 713.1805 | 1.4805 | 790.154 | 1.4903 | 993.1516 |
| 1.9335 | 576.6728 | 1.4936 | 713.4438 | 1.8095 | 674.836 | 1.5764 | 995.5088 |
| 2.2647 | 533.3138 | 1.8248 | 686.9869 | 2.1386 | 548.622 | 1.9076 | 959.1524 |
| 2.596 | 489.6236 | 2.156 | 643.3836 | 2.4675 | 473.1292 | 2.2388 | 896.4296 |
| 2.9271 | 445.8845 | 2.4872 | 598.2051 | 2.7965 | 412.2097 | 2.57 | 822.3504 |
| 3.2583 | 409.1873 | 2.8184 | 554.5164 | 3.1256 | 370.2771 | 2.9012 | 764.2773 |
| 3.5895 | 377.2928 | 3.1496 | 515.1858 | 3.4546 | 333.0229 | 3.2324 | 695.8102 |
| 3.9207 | 351.778 | 3.4808 | 480.6596 | 3.7836 | 307.3465 | 3.5635 | 646.1151 |
| 4.2519 | 330.2087 | 3.812 | 451.4683 | 4.1126 | 289.231 | 3.8948 | 604.6989 |
| 4.5831 | 314.0207 | 4.1431 | 433.1013 | 4.4416 | 275.025 | 4.226 | 564.0626 |
| 4.9143 | 299.5968 | 4.4744 | 411.7863 | 4.7706 | 264.5642 | 4.5571 | 519.03 |
| 5.2455 | 292.3239 | 4.8055 | 391.2814 | 5.0996 | 259.4523 | 4.8883 | 474.0193 |
| 5.5767 | 282.0634 | 5.1367 | 373.7882 | 5.4287 | 254.011 | 5.2195 | 434.0626 |
| 5.9079 | 275.354 | 5.4679 | 359.0372 | 5.7577 | 247.8774 | 5.5507 | 404.7392 |
| 6.2391 | 268.5133 | 5.7991 | 348.1859 | 6.0867 | 243.4336 | 5.882 | 380.45 |
| 6.5703 | 264.7179 | 6.1303 | 334.5741 | 6.4157 | 247.993 | 6.2131 | 357.6116 |
| 6.9015 | 260.3415 | 6.4615 | 323.0586 | 6.7447 | 242.0013 | 6.5443 | 336.3872 |
| 7.2326 | 257.5439 | 6.7927 | 310.3872 | 7.0737 | 235.7566 | 6.8755 | 315.1651 |
| 7.5638 | 253.837 | 7.1239 | 304.3122 | 7.4028 | 231.8409 | 7.2067 | 302.622 |
| 7.895 | 249.0747 | 7.4551 | 300.5565 | 7.7317 | 230.9326 | 7.5379 | 298.9886 |
| 8.2262 | 244.2835 | 7.7863 | 290.2953 | 8.0608 | 225.2588 | 7.869 | 284.283 |
| 8.5574 | 235.0071 | 8.1175 | 284.685 | 8.3898 | 216.4254 | 8.2008 | 287.5706 |
| 8.8886 | 233.8358 | 8.4486 | 271.8231 | 8.7188 | 216.1209 | 8.532 | 291.0991 |
| 9.2198 | 231.7573 | 8.7799 | 272.5655 | 9.0478 | 208.4633 | 8.8632 | 290.8645 |
| 9.551 | 230.3078 | 9.1111 | 266.6608 | 9.3768 | 208.2124 | 9.1944 | 281.9811 |
| 9.8822 | 228.2399 | 9.4422 | 264.7067 | 9.7058 | 207.6375 | 9.5256 | 282.3241 |
| / | / | 9.7734 | 267.3542 | / | / | 9.8568 | 277.002 |

Table 3 Permanent strain for samples with CD=0.95 under the confining pressure of 40 kPa

| Loading cycles | Permanent strain (%) | | | | | | |
| --- | --- | --- | --- | --- | --- | --- | --- |
|  | 5% | 10% | 20% | 30% | 40% | 60% | 80% |
| 0 | 0.000 | 0.000 | 0.000 | 0.000 | 0.000 | 0.000 | 0.000 |
| 10 | 0.006 | 0.010 | 0.013 | 0.013 | 0.015 | 0.031 | 0.038 |
| 20 | 0.010 | 0.014 | 0.018 | 0.018 | 0.019 | 0.055 | 0.061 |
| 30 | 0.012 | 0.016 | 0.020 | 0.021 | 0.022 | 0.068 | 0.080 |
| 40 | 0.014 | 0.019 | 0.022 | 0.023 | 0.024 | 0.079 | 0.098 |
| 50 | 0.016 | 0.022 | 0.023 | 0.024 | 0.026 | 0.089 | 0.114 |
| 60 | 0.017 | 0.024 | 0.024 | 0.026 | 0.028 | 0.098 | 0.130 |
| 70 | 0.019 | 0.026 | 0.026 | 0.027 | 0.029 | 0.106 | 0.144 |
| 80 | 0.020 | 0.027 | 0.027 | 0.029 | 0.030 | 0.113 | 0.158 |
| 90 | 0.022 | 0.028 | 0.027 | 0.030 | 0.032 | 0.120 | 0.171 |
| 100 | 0.022 | 0.029 | 0.028 | 0.031 | 0.033 | 0.127 | 0.184 |
| 200 | 0.024 | 0.035 | 0.035 | 0.040 | 0.039 | 0.183 | 0.256 |
| 300 | 0.025 | 0.037 | 0.040 | 0.048 | 0.045 | 0.227 | 0.445 |
| 400 | 0.026 | 0.038 | 0.044 | 0.053 | 0.050 | 0.263 | 0.643 |
| 500 | 0.027 | 0.040 | 0.047 | 0.057 | 0.054 | 0.296 | 0.849 |
| 600 | 0.028 | 0.041 | 0.049 | 0.059 | 0.058 | 0.325 | 1.076 |
| 700 | 0.028 | 0.042 | 0.051 | 0.062 | 0.062 | 0.351 | 1.328 |
| 800 | 0.029 | 0.043 | 0.052 | 0.064 | 0.066 | 0.376 | 1.622 |
| 900 | 0.029 | 0.043 | 0.054 | 0.066 | 0.069 | 0.400 | 1.980 |
| 1000 | 0.030 | 0.044 | 0.055 | 0.068 | 0.072 | 0.422 | 2.398 |
| 1400 | 0.031 | 0.046 | 0.059 | 0.074 | 0.082 | 0.502 | 2.925 |
| 1800 | 0.033 | 0.047 | 0.062 | 0.078 | 0.090 | 0.571 | 4.662 |
| 2200 | 0.034 | 0.049 | 0.065 | 0.082 | 0.097 | 0.635 | 7.004 |
| 2600 | 0.034 | 0.051 | 0.067 | 0.085 | 0.104 | 0.692 | 13.760 |
| 3000 | 0.035 | 0.052 | 0.069 | 0.088 | 0.109 | 0.747 |  |
| 3400 | 0.036 | 0.053 | 0.070 | 0.090 | 0.114 | 0.798 |  |
| 3800 | 0.036 | 0.054 | 0.072 | 0.092 | 0.118 | 0.845 |  |
| 4200 | 0.037 | 0.055 | 0.073 | 0.095 | 0.122 | 0.892 |  |
| 4600 | 0.037 | 0.056 | 0.074 | 0.096 | 0.126 | 0.936 |  |
| 5000 | 0.038 | 0.057 | 0.076 | 0.098 | 0.130 | 0.979 |  |
| 5500 | 0.038 | 0.058 | 0.077 | 0.100 | 0.135 | 1.031 |  |
| 6000 | 0.039 | 0.059 | 0.078 | 0.102 | 0.139 | 1.083 |  |
| 6500 | 0.039 | 0.059 | 0.079 | 0.104 | 0.142 | 1.133 |  |
| 7000 | 0.039 | 0.060 | 0.080 | 0.106 | 0.146 | 1.183 |  |
| 7500 | 0.040 | 0.061 | 0.082 | 0.108 | 0.150 | 1.232 |  |
| 8000 | 0.040 | 0.061 | 0.083 | 0.109 | 0.153 | 1.280 |  |
| 8500 | 0.040 | 0.062 | 0.084 | 0.111 | 0.157 | 1.329 |  |
| 9000 | 0.040 | 0.063 | 0.085 | 0.112 | 0.160 | 1.378 |  |
| 9500 | 0.040 | 0.063 | 0.085 | 0.114 | 0.163 | 1.427 |  |
| 10000 | 0.041 | 0.063 | 0.086 | 0.115 | 0.166 | 1.458 |  |

Table 4 Permanent strain for samples with CD=0.95 under the confining pressure of 60 kPa

| Loading cycles | Permanent strain (%) | | | | | | |
| --- | --- | --- | --- | --- | --- | --- | --- |
|  | 5% | 10% | 20% | 30% | 40% | 60% | 80% |
| 0 | 0 | 0 | 0 | 0 | 0 | 0 | 0 |
| 10 | 0.008 | 0.011 | 0.014 | 0.017 | 0.010 | 0.038 | 0.145 |
| 20 | 0.011 | 0.012 | 0.019 | 0.021 | 0.013 | 0.053 | 0.200 |
| 30 | 0.012 | 0.013 | 0.022 | 0.023 | 0.015 | 0.065 | 0.240 |
| 40 | 0.014 | 0.015 | 0.025 | 0.025 | 0.017 | 0.075 | 0.274 |
| 50 | 0.015 | 0.017 | 0.027 | 0.026 | 0.019 | 0.084 | 0.303 |
| 60 | 0.017 | 0.018 | 0.029 | 0.028 | 0.021 | 0.092 | 0.329 |
| 70 | 0.018 | 0.019 | 0.031 | 0.029 | 0.022 | 0.099 | 0.353 |
| 80 | 0.019 | 0.020 | 0.033 | 0.030 | 0.023 | 0.106 | 0.376 |
| 90 | 0.020 | 0.021 | 0.034 | 0.031 | 0.025 | 0.113 | 0.396 |
| 100 | 0.021 | 0.022 | 0.035 | 0.032 | 0.026 | 0.119 | 0.416 |
| 200 | 0.024 | 0.027 | 0.042 | 0.035 | 0.034 | 0.169 | 0.571 |
| 300 | 0.026 | 0.028 | 0.048 | 0.040 | 0.041 | 0.206 | 0.687 |
| 400 | 0.027 | 0.029 | 0.053 | 0.044 | 0.047 | 0.238 | 0.801 |
| 500 | 0.029 | 0.031 | 0.056 | 0.048 | 0.052 | 0.266 | 0.916 |
| 600 | 0.029 | 0.032 | 0.059 | 0.051 | 0.056 | 0.292 | 1.028 |
| 700 | 0.030 | 0.033 | 0.061 | 0.053 | 0.060 | 0.315 | 1.142 |
| 800 | 0.030 | 0.034 | 0.062 | 0.056 | 0.063 | 0.337 | 1.258 |
| 900 | 0.031 | 0.034 | 0.064 | 0.058 | 0.067 | 0.357 | 1.380 |
| 1000 | 0.031 | 0.035 | 0.066 | 0.060 | 0.070 | 0.377 | 1.504 |
| 1400 | 0.032 | 0.036 | 0.070 | 0.066 | 0.080 | 0.447 | 2.022 |
| 1800 | 0.033 | 0.038 | 0.074 | 0.071 | 0.089 | 0.509 | 2.544 |
| 2200 | 0.034 | 0.040 | 0.077 | 0.075 | 0.096 | 0.570 | 3.093 |
| 2600 | 0.035 | 0.041 | 0.079 | 0.079 | 0.103 | 0.628 | 3.736 |
| 3000 | 0.036 | 0.042 | 0.081 | 0.082 | 0.109 | 0.686 | 4.439 |
| 3400 | 0.036 | 0.043 | 0.082 | 0.084 | 0.113 | 0.743 | 5.148 |
| 3800 | 0.037 | 0.044 | 0.084 | 0.087 | 0.118 | 0.799 | 5.918 |
| 4200 | 0.037 | 0.045 | 0.085 | 0.089 | 0.122 | 0.853 | 6.739 |
| 4600 | 0.038 | 0.046 | 0.087 | 0.092 | 0.126 | 0.907 | 7.658 |
| 5000 | 0.038 | 0.047 | 0.088 | 0.094 | 0.129 | 0.959 | 8.683 |
| 5500 | 0.039 | 0.048 | 0.089 | 0.096 | 0.134 | 1.020 | 9.876 |
| 6000 | 0.039 | 0.048 | 0.090 | 0.099 | 0.138 | 1.078 | 10.978 |
| 6500 | 0.040 | 0.049 | 0.091 | 0.101 | 0.142 | 1.133 | 12.036 |
| 7000 | 0.040 | 0.050 | 0.092 | 0.102 | 0.146 | 1.187 | 13.042 |
| 7500 | 0.040 | 0.050 | 0.093 | 0.104 | 0.149 | 1.240 |  |
| 8000 | 0.040 | 0.051 | 0.094 | 0.105 | 0.153 | 1.293 |  |
| 8500 | 0.041 | 0.051 | 0.095 | 0.107 | 0.157 | 1.345 |  |
| 9000 | 0.041 | 0.052 | 0.096 | 0.108 | 0.160 | 1.395 |  |
| 9500 | 0.041 | 0.052 | 0.097 | 0.109 | 0.164 | 1.442 |  |
| 10000 | 0.042 | 0.053 | 0.097 | 0.111 | 0.166 | 1.511 |  |

Table 5 Permanent strain for samples with CD=1.00 under the confining pressure of 40 kPa

| Loading cycles | Permanent strain (%) | | | | | | |
| --- | --- | --- | --- | --- | --- | --- | --- |
|  | 5% | 10% | 20% | 30% | 40% | 60% | 80% |
| 0 | 0 | 0 | 0 | 0 | 0 | 0 | 0 |
| 10 | 0.008 | 0.014 | 0.011 | 0.011 | 0.026 | 0.018 | 0.030 |
| 20 | 0.013 | 0.018 | 0.013 | 0.014 | 0.030 | 0.025 | 0.053 |
| 30 | 0.017 | 0.020 | 0.015 | 0.016 | 0.033 | 0.031 | 0.073 |
| 40 | 0.019 | 0.021 | 0.016 | 0.018 | 0.035 | 0.036 | 0.092 |
| 50 | 0.021 | 0.022 | 0.016 | 0.019 | 0.037 | 0.040 | 0.110 |
| 60 | 0.022 | 0.023 | 0.017 | 0.020 | 0.038 | 0.044 | 0.127 |
| 70 | 0.024 | 0.024 | 0.018 | 0.022 | 0.039 | 0.048 | 0.143 |
| 80 | 0.026 | 0.025 | 0.018 | 0.022 | 0.041 | 0.051 | 0.159 |
| 90 | 0.027 | 0.025 | 0.019 | 0.023 | 0.042 | 0.054 | 0.175 |
| 100 | 0.028 | 0.026 | 0.019 | 0.024 | 0.043 | 0.057 | 0.191 |
| 200 | 0.036 | 0.029 | 0.023 | 0.030 | 0.049 | 0.082 | 0.331 |
| 300 | 0.039 | 0.032 | 0.026 | 0.034 | 0.054 | 0.100 | 0.456 |
| 400 | 0.041 | 0.034 | 0.029 | 0.038 | 0.058 | 0.116 | 0.580 |
| 500 | 0.042 | 0.036 | 0.032 | 0.041 | 0.061 | 0.130 | 0.708 |
| 600 | 0.043 | 0.037 | 0.034 | 0.044 | 0.064 | 0.143 | 0.846 |
| 700 | 0.044 | 0.039 | 0.036 | 0.046 | 0.066 | 0.154 | 0.993 |
| 800 | 0.044 | 0.040 | 0.038 | 0.048 | 0.068 | 0.164 | 1.150 |
| 900 | 0.045 | 0.041 | 0.040 | 0.050 | 0.070 | 0.175 | 1.318 |
| 1000 | 0.046 | 0.042 | 0.042 | 0.052 | 0.071 | 0.184 | 1.499 |
| 1400 | 0.048 | 0.045 | 0.049 | 0.058 | 0.077 | 0.218 | 2.354 |
| 1800 | 0.049 | 0.047 | 0.054 | 0.062 | 0.081 | 0.250 | 3.745 |
| 2200 | 0.050 | 0.049 | 0.059 | 0.066 | 0.084 | 0.279 | 5.538 |
| 2600 | 0.052 | 0.050 | 0.063 | 0.069 | 0.086 | 0.308 | 7.739 |
| 3000 | 0.052 | 0.052 | 0.069 | 0.072 | 0.089 | 0.337 |  |
| 3400 | 0.053 | 0.053 | 0.071 | 0.075 | 0.090 | 0.364 |  |
| 3800 | 0.054 | 0.054 | 0.073 | 0.077 | 0.092 | 0.393 |  |
| 4200 | 0.054 | 0.055 | 0.075 | 0.079 | 0.093 | 0.422 |  |
| 4600 | 0.055 | 0.056 | 0.077 | 0.081 | 0.095 | 0.451 |  |
| 5000 | 0.056 | 0.057 | 0.079 | 0.082 | 0.096 | 0.481 |  |
| 5500 | 0.056 | 0.058 | 0.081 | 0.084 | 0.097 | 0.519 |  |
| 6000 | 0.057 | 0.059 | 0.083 | 0.086 | 0.099 | 0.559 |  |
| 6500 | 0.057 | 0.060 | 0.085 | 0.088 | 0.100 | 0.598 |  |
| 7000 | 0.058 | 0.060 | 0.087 | 0.090 | 0.101 | 0.637 |  |
| 7500 | 0.058 | 0.061 | 0.088 | 0.092 | 0.102 | 0.679 |  |
| 8000 | 0.059 | 0.061 | 0.090 | 0.093 | 0.102 | 0.723 |  |
| 8500 | 0.059 | 0.062 | 0.092 | 0.095 | 0.103 | 0.770 |  |
| 9000 | 0.060 | 0.062 | 0.093 | 0.097 | 0.104 | 0.819 |  |
| 9500 | 0.060 | 0.062 | 0.094 | 0.098 | 0.105 | 0.870 |  |
| 10000 | 0.060 | 0.063 | 0.095 | 0.100 | 0.106 | 0.923 |  |

Table 6 Permanent strain for samples with CD=1.00 under the confining pressure of 60 kPa

| Loading cycles | Permanent strain (%) | | | | | | |
| --- | --- | --- | --- | --- | --- | --- | --- |
|  | 5% | 10% | 20% | 30% | 40% | 60% | 80% |
| 0 | 0 | 0 | 0 | 0 | 0 | 0 | 0 |
| 10 | 0.015 | 0.022 | 0.012 | 0.011 | 0.029 | 0.031 | 0.031 |
| 20 | 0.023 | 0.026 | 0.016 | 0.014 | 0.034 | 0.044 | 0.054 |
| 30 | 0.027 | 0.028 | 0.018 | 0.016 | 0.038 | 0.053 | 0.075 |
| 40 | 0.029 | 0.029 | 0.020 | 0.018 | 0.041 | 0.061 | 0.094 |
| 50 | 0.030 | 0.031 | 0.022 | 0.019 | 0.043 | 0.069 | 0.113 |
| 60 | 0.032 | 0.032 | 0.023 | 0.020 | 0.045 | 0.075 | 0.131 |
| 70 | 0.033 | 0.033 | 0.025 | 0.022 | 0.047 | 0.081 | 0.148 |
| 80 | 0.034 | 0.034 | 0.026 | 0.023 | 0.049 | 0.086 | 0.165 |
| 90 | 0.035 | 0.035 | 0.027 | 0.024 | 0.050 | 0.092 | 0.182 |
| 100 | 0.036 | 0.036 | 0.028 | 0.025 | 0.051 | 0.097 | 0.198 |
| 200 | 0.041 | 0.041 | 0.036 | 0.028 | 0.061 | 0.133 | 0.348 |
| 300 | 0.044 | 0.044 | 0.041 | 0.033 | 0.068 | 0.163 | 0.488 |
| 400 | 0.047 | 0.047 | 0.046 | 0.037 | 0.074 | 0.189 | 0.630 |
| 500 | 0.049 | 0.049 | 0.049 | 0.041 | 0.078 | 0.212 | 0.774 |
| 600 | 0.050 | 0.051 | 0.053 | 0.045 | 0.082 | 0.233 | 0.923 |
| 700 | 0.052 | 0.053 | 0.056 | 0.049 | 0.085 | 0.252 | 1.079 |
| 800 | 0.053 | 0.054 | 0.058 | 0.052 | 0.088 | 0.270 | 1.244 |
| 900 | 0.054 | 0.056 | 0.061 | 0.055 | 0.090 | 0.286 | 1.427 |
| 1000 | 0.055 | 0.057 | 0.063 | 0.057 | 0.093 | 0.302 | 1.623 |
| 1400 | 0.058 | 0.061 | 0.070 | 0.066 | 0.099 | 0.360 | 2.609 |
| 1800 | 0.060 | 0.064 | 0.076 | 0.074 | 0.105 | 0.413 | 4.204 |
| 2200 | 0.062 | 0.066 | 0.081 | 0.080 | 0.109 | 0.463 |  |
| 2600 | 0.063 | 0.069 | 0.085 | 0.086 | 0.112 | 0.509 |  |
| 3000 | 0.065 | 0.070 | 0.088 | 0.091 | 0.115 | 0.556 |  |
| 3400 | 0.066 | 0.072 | 0.091 | 0.095 | 0.117 | 0.607 |  |
| 3800 | 0.067 | 0.073 | 0.093 | 0.099 | 0.119 | 0.656 |  |
| 4200 | 0.068 | 0.075 | 0.096 | 0.103 | 0.121 | 0.704 |  |
| 4600 | 0.068 | 0.076 | 0.098 | 0.106 | 0.123 | 0.753 |  |
| 5000 | 0.069 | 0.077 | 0.100 | 0.109 | 0.125 | 0.801 |  |
| 5500 | 0.070 | 0.078 | 0.102 | 0.113 | 0.127 | 0.861 |  |
| 6000 | 0.071 | 0.079 | 0.104 | 0.116 | 0.129 | 0.919 |  |
| 6500 | 0.072 | 0.080 | 0.105 | 0.119 | 0.130 | 0.977 |  |
| 7000 | 0.073 | 0.081 | 0.107 | 0.122 | 0.132 | 1.036 |  |
| 7500 | 0.073 | 0.082 | 0.109 | 0.125 | 0.133 | 1.095 |  |
| 8000 | 0.074 | 0.083 | 0.110 | 0.128 | 0.135 | 1.152 |  |
| 8500 | 0.074 | 0.084 | 0.112 | 0.130 | 0.137 | 1.211 |  |
| 9000 | 0.075 | 0.084 | 0.114 | 0.133 | 0.139 | 1.269 |  |
| 9500 | 0.075 | 0.085 | 0.115 | 0.136 | 0.141 | 1.328 |  |
| 10000 | 0.076 | 0.085 | 0.117 | 0.139 | 0.143 | 1.373 |  |

Table 7 The relationship between comprehensive evaluation coefficient α and CSR.

| CSR (%) | CD=0.95 | | CD=1.00 | |
| --- | --- | --- | --- | --- |
|  | 40 kPa | 60 kPa | 40 kPa | 60 kPa |
| 5 | 0.002 | 0.002 | 0.003 | 0.004 |
| 10 | 0.017 | 0.015 | 0.020 | 0.028 |
| 20 | 0.030 | 0.030 | 0.056 | 0.048 |
| 30 | 0.051 | 0.051 | 0.087 | 0.074 |
| 40 | 0.083 | 0.082 | 0.074 | 0.095 |
| 60 | 0.478 | 0.326 | 0.218 | 0.317 |
| 80 | 3.922 | 1.396 | 2.581 | 2.501 |

Table 8 The relationship between comprehensive evaluation coefficient *β* and CSR.

| CSR (%) | CD=0.95 | | CD=1.00 | |
| --- | --- | --- | --- | --- |
|  | 40 kPa | 60 kPa | 40 kPa | 60 kPa |
| 5 | 2.01 | 2.01 | 1.91 | 1.89 |
| 10 | 1.37 | 1.41 | 1.39 | 1.35 |
| 20 | 1.04 | 1.04 | 1 | 0.99 |
| 30 | 1 | 0.97 | 0.93 | 0.89 |
| 40 | 0.9 | 0.88 | 0.94 | 0.94 |
| 60 | 0.5 | 0.52 | 0.48 | 0.5 |
| 80 | -0.32 | -0.34 | -0.73 | -0.54 |
